# Effect of Obesity on the Expression of Nutrient Receptors and Satiety Hormones in the Human Colon

**DOI:** 10.3390/nu13041271

**Published:** 2021-04-13

**Authors:** Lucas Baumard, Zsa Zsa R. M. Weerts, Ad A. M. Masclee, Daniel Keszthelyi, Adina T. Michael-Titus, Madusha Peiris

**Affiliations:** 1 Centre for Neuroscience, Surgery and Trauma, Blizard Institute, Barts and The London School of Medicine & Dentistry, Queen Mary University of London, London E1 2AT, UK; l.baumard@qmul.ac.uk (L.B.); a.t.michael-titus@qmul.ac.uk (A.T.M.-T.); 2 Division of Gastroenterology and Hepatology, Department of Internal Medicine, School for Nutrition and Translational Research in Metabolism, Maastricht University Medical Centre, 6229 Maastricht, The Netherlands; z.weerts@maastrichtuniversity.nl (Z.Z.R.M.W.); a.masclee@mumc.nl (A.A.M.M.); daniel.keszthelyi@maastrichtuniversity.nl (D.K.)

**Keywords:** nutrient sensing, enteroendocrine cells (EECs), appetite regulation, G-protein coupled receptors (GPCRs), obesity

## Abstract

Background: Receptors located on enteroendocrine cells (EECs) of the colon can detect nutrients in the lumen. These receptors regulate appetite through a variety of mechanisms, including hormonal and neuronal signals. We assessed the effect of obesity on the expression of these G-protein coupled receptors (GPCRs) and hormones at both mRNA and protein level. Methods: qPCR and immunohistochemistry were used to examine colonic tissue from cohorts of patients from the Netherlands (proximal and sigmoid tissue) and the United Kingdom (tissue from across the colon) and patients were grouped by body mass index (BMI) value (BMI < 25 and BMI ≥ 25). Results: The mRNA expression of the hormones/signaling molecules serotonin, glucagon, peptide YY (PYY), CCK and somatostatin were not significantly different between BMI groups. GPR40 mRNA expression was significantly increased in sigmoid colon samples in the BMI ≥ 25 group, but not proximal colon. GPR41, GPR109a, GPR43, GPR120, GPRC6A, and CaSR mRNA expression were unaltered between low and high BMI. At the protein level, serotonin and PYY containing cell numbers were similar in high and low BMI groups. Enterochromaffin cells (EC) showed high degree of co-expression with amino acid sensing receptor, CaSR while co-expression with PYY containing L-cells was limited, regardless of BMI. Conclusions: While expression of medium/long chain fatty acid receptor GPR40 was increased in the sigmoid colon of the high BMI group, expression of other nutrient sensing GPCRs, and expression profiles of EECs involved in peripheral mechanisms of appetite regulation were unchanged. Collectively, these data suggest that in human colonic tissue, EEC and nutrient-sensing receptor expression profiles are not affected despite changes to BMI.

## 1. Introduction

Obesity is a significant and growing health issue facing the Western world [1,2]. Obese adults are more susceptible to a wide range of comorbidities, including type 2 diabetes [3], metabolic disorders and abnormalities such as dyslipidaemia [4], cardiovascular disease [5]—including hypertension [6], stroke [7]—and a variety of cancers [8].

Appetite is regulated to balance energy intake and expenditure by the release of hunger and satiety signals [9]. Appetite regulation can begin before food enters the body; the cephalic phase is initiated by sights and smells of food and by environmental effects such as time of day [10]. Both mechanical and hormonal signals regulate food intake via central control from brain centres of appetite regulation, including the solitary tract nucleus in the brain stem [11,12]. Potent satiety hormones such as PYY and glucagon-like peptide 1 (GLP-1) are released from the small intestine to the colon, in response to dietary nutrients [13]. The colon is the main source of peripheral serotonin [14] and holds the highest number and diversity of microbiota, with bacterial by-products such as short chain fatty acids (SCFA) playing a role in nutrient sensing and obesity. Hormones such as PYY, CCK, and GLP-1 are released from the colon and can enter the circulation [15,16]. PYY can stimulate neurons within the hypothalamic arcuate nucleus: orexigenic neurons co-expresses agouti-related peptide (AgRP) and neuropeptide Y (NPY); anorexigenic neurons release pro-opiomelanocortin (POMC) and cocaine- and amphetamine-regulated transcript (CART) [9]. CCK can act on vagal fibres innervating the brain stem to alter gut motility, secretions from the pancreas and gall bladder activation [9].

Enteroendocrine cells (EECs) are a subset of epithelial cells expressed throughout gastrointestinal tissues and are classified according to their hormone/peptide content [17,18]. EECs express nutrient-sensing receptors and respond to luminal contents, leading to the release of anorectic hormones and mediators that act on the vagus nerve and hypothalamus, resulting in long-term satiation [18,19]. L cells, an EEC sub-type critical for appetite regulation, co-secrete the potent anorectic hormones PYY and GLP-1, and are highly expressed in the colon [20,21]. PYY acts on receptors within the hypothalamus to inhibit the release of the orexigenic peptide neuropeptide Y (NPY) [22]. These same receptors are also found on peripheral vagal afferents [10] and are likely to drive PYY’s role in regulating gastric acid secretion and gastrointestinal motility [22]. GLP-1 receptors are also found within the hypothalamus and brain stem, where their activation reduces food intake [23]. In obesity, circulating levels of PYY and GLP-1 are decreased [24,25]. Enterochromaffin (EC) cells, a further population of EECs, secrete 95% of the body’s serotonin in response to carbohydrate-rich foods and SCFAs [26]. Centrally released serotonin plays an important role in modulating food cravings and mood [27,28] whilst serotonin from the colon and periphery can activate small intestinal and colonic vagal afferent nerves to modulate gastrointestinal secretion and motility [14,28]. Drugs that can act as serotonin receptor agonists centrally have been shown to reduce weight gain in mice and caloric intake in lean and obese humans via the hypothalamic melanocortin system [29]. Serotonin is synthesised from tryptophan by the enzyme tryptophan hydroxylase 1 (TPH1), in a rate limiting process [30]. Cholecystokinin (CCK), released by I cells [31], strongly reduces food intake [32], and CCK administration reduces food intake in obese patients [33]. Finally, somatostatin is expressed in D cells [34] and inhibits the release of anorectic hormones like GLP-1 and PYY [35,36], demonstrating a critical cellular control mechanism. Collectively, various EECs are required for the normal processes of appetite regulation.

Importantly, to activate EECs and induce release of hormones/peptides, nutrients bind to and activate nutrient sensing G-protein coupled receptors (GPCRs) expressed on EECs [37]. SCFAs can bind to GPCRs via GPR41 [38], GPR43 [39], and GPR109a [40], medium/long chain fatty acids (M/LCFA) bind to GPR40 [41] and GPR120 [42] and amino acids bind to CaSR [43] and GPRC6a [44]. These receptors are expressed throughout the gastrointestinal tract, including the colon and rectum, in both mice and humans [45]. L-cells express the SCFA receptors GPR43, GPR41 [46], and CaSR [47] and colonic ECs have been shown to express GPR119, GPR120, GPR41, and GPR43 [48]. We have previously shown that agonist action at GPR41, GPR40, and GPR119 induces colonic EEC activation [49] and, importantly, that stimulation of GPR84 increases the release of GLP-1, PYY, and serotonin, demonstrating functional modulation of L and EC cells [45].

There is evidence to suggest that obesity alters peripheral pathways of appetite regulation. The mRNA expression and protein levels of GPR120 are decreased in the visceral adipose tissue of obese individuals [50], while GPR120 knockout mice demonstrate an obese phenotype [51]. mRNA expression of GPR41, GPR43, GPR40, and GPR120 increased in diet-induced obese mice compared to mice on a normal diet [49]. Additionally, post-prandial release of hormones is altered, with lower circulating levels of PYY and GLP-1 reported in obesity [9,52], with levels remaining low after 10 weeks of sustained weight loss [53]. Collectively, these data suggest that obesity may alter peripheral mechanisms of appetite regulation.

The aim of this study was to assess expression profiles of EECs and nutrient-sensing GPCRs, to determine if weight gain alters the physiology of nutrient sensing at the cellular level, particularly in the colon.

## 2. Materials and Methods

### 2.1. Human Samples

Tissue specimens were collected in two centres: The Royal London Hospital, in London, UK, and the Maastricht University Medical Centre (MUMC), in Maastricht, the Netherlands.

In London, non-inflamed, non-cancerous (morphologically normal), full thickness, and mucosal samples were taken from the ascending, transverse, and descending colon of patients (*n* = 30) undergoing gastrointestinal cancer surgery.

In Maastricht, biopsies were taken from the right sided proximal and the sigmoid colon of patients with irritable bowel syndrome (*n* = 30) participating in the Maastricht IBS cohort study and undergoing routine colonoscopy. In addition, biopsies were taken from the sigmoid colon of healthy controls (*n* = 7) participating in an interventional study (placebo group). Inclusion and exclusion criteria of the Maastricht IBS cohort and the healthy control group are described elsewhere [54,55]. Biopsies were placed in Eppendorf tubes immediately and fresh frozen in liquid nitrogen to be stored at −80 °C until RNA isolation.

All participants gave written informed consent prior to inclusion. The studies were approved by the East London and The City HA Local Research Ethics Committee (NREC 09/H0704/2) and the University of Maastricht Medical Ethics Committee, respectively, and were performed in compliance with the revised Declaration of Helsinki (64th WMA General Assembly, Fortaleza, Brazil, 2013). All samples collected were from fasted subjects with tissue collection occurring in the morning. The study is registered in the US National Library of Medicine (http://www.clinicaltrials.gov, NCT00775060, 2008, accessed on 5 March 2021). Patient and healthy control characteristics are presented in Supplementary Tables S1 and S2, with the distribution of participant BMI from both research locations shown in Supplementary Graphs S1 and S2.

### 2.2. Immunohistochemistry

Patient samples used for immunohistochemistry were obtained from the Royal London Hospital as previously described. Full thickness samples were fixed in 4% paraformaldehyde/phosphate buffered saline (PBS) at 4 °C overnight. Tissue was cryoprotected in 30% sucrose/PBS, then mounted in OCT embedding compound for cutting and subsequent storage at −80 °C.

Cut tissue sections (10 μm) were washed with PBS, blocked with Trident Universal Protein Blocking Reagent (animal serum free) (Insight Biotechnology limited GTX 30963) and primary antibody was applied (Supplementary Table S3) for 18 h at 4 °C. Tissue was then washed in PBS and incubated for 1 h with species-specific Alexa Fluor conjugated secondary antibodies (Supplementary Table S4 (1:400)). Slides were cover-slipped with a mounting medium containing diamidino-2-phenylindole (DAPI) (VECTASHIELD, Vector laboratories H-1500). Negative controls were obtained by omitting the primary antibody. Sections were visualised and imaged on an epifluorescence microscope (Leica DM4000 Epi-Fluorescence Microscope) and images were acquired on a monochrome CCD digital camera system (Leica DFC365) using Metamorph imaging system software. Images were analysed using ImageJ (Rasband, W.S., ImageJ, U. S. National Institutes of Health, Bethesda, MD, USA, https://imagej.nih.gov/ij/, 1997–2018, accessed on 5 March 2021).

Cell counts were performed on stained sections from a colonic region and positively stained cells were manually counted. Counts were performed blinded to patient data, including BMI. A minimum of 10 field of views (FOV) were taken in each section, the number of cells and crypts were counted and the average value calculated for cells/crypt for each field of view and patient. Cells were counted based on the inclusion and exclusion criteria listed in Table 1.

### 2.3. Gene Expression Studies

Quantitative real-time reverse transcriptase PCR (RT-PCR) was used to assess the relative expression of nutrient GPCRs and hormone/peptides in human colonic tissue (Supplementary Table S6 for patient and tissue details).

Mucosal samples were stored in RNALater (Qiagen, Manchester, UK) at −80 °C prior to gene expression experiments. RNA was extracted from tissues using a RNeasy Mini kit (Qiagen). RNA quantity and quality were assessed using a NanoDrop machine. RNA was reverse transcribed into cDNA using the Quantitech RT kit (Qiagen) or the High-Capacity cDNA Reverse Transcription Kit with RNase inhibitor (Applied biosystems, Thermo Fisher Scientific) for the London and Maastricht samples, respectively.

cDNA samples from Maastricht were reverse transcribed using MultiScribe Reverse Transcriptase (ThermoFischer Scientific, Waltham, MA, USA). These samples were run on TaqMan PCR (Thermofisher TaqMan Array Micro Fluidic card Cat# 4342249) using a ViiA7 machine. For each patient, the relative gene expression of genes of interest and 2 positive controls (18s ribosomal RNA (18s) and glyceraldehyde-3-phosphate dehydrogenase (GAPDH)) were calculated for each plate. The relative expression of mRNA from the sigmoid and proximal colon was plotted separately against BMI. BMI values were used to divide patients into normal weight patients (BMI < 25) and obese/overweight patients (BMI ≥ 25). We also used regression analysis (Pearson r correlation and simple linear regression) with BMI as the co-variant, and similar results were obtained (data not shown). The patient group of BMI < 25 are all those patients below or equal to 24.9 BMI. Sigmoidal and proximal colon samples were analysed separately. Supplementary Table S1 shows the number of samples for each gene and colonic location and Supplementary Table S2 the BMI distribution of these patients.

For samples from the Royal London Hospital, SYBR green primers were purchased from Qiagen (Supplementary Table S5). Target gene expression was determined relative to the endogenous control, 18s ribosomal RNA, using the comparative cycle threshold method on an Applied Biosystems StepOnePLus real-time PCR system thermal cycling block.

### 2.4. Statistical Analysis

Data is expressed as mean ± SEM. Statistical analysis for mRNA expression and cell counting was performed using unpaired *t* tests (Mann-Whitney test) (GraphPad Prism, V.8, GraphPad Software Inc, San Diego, CA, USA), with *p* < 0.05 considered statistically significant.

## 3. Results

### 3.1. The mRNA Expression of Hormones and Peptides Involved in Appetite Regulation Is Unchanged between Healthy BMI (<25) and Overweight/Obese BMI (≥25)

**Figure 1:**
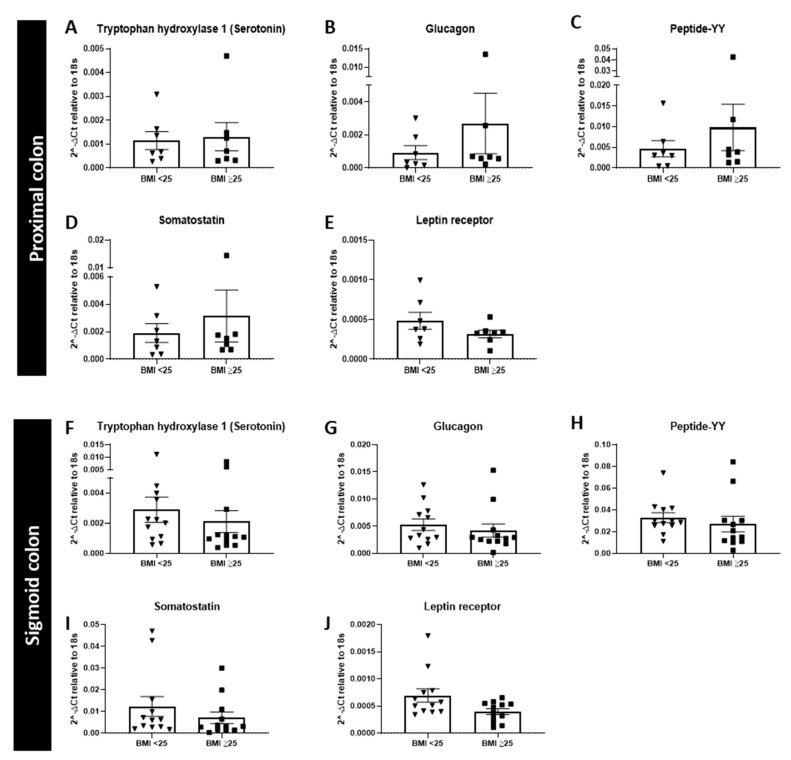
Relative mRNA expression of hormones and hormone receptors involved in satiety in the proximal and sigmoid colon. mRNA expression (relative to 18s) of hormone markers in the proximal (**A**–**E**), (body mass index (BMI) < 25 *n* = 7, BMI ≥ 25 *n* = 7), and sigmoid colon (**F**–**J**), (BMI < 25 *n* = 12, BMI ≥ 25 *n* = 12).

In the proximal colon, there was no significant change in the expression of tryptophan hydroxylase 1 (TPH1), glucagon, PYY or somatostatin, between the healthy BMI (<25) or overweight/obese BMI group (≥25) (Figure 1A–E). We also assessed the expression of the leptin receptor and observed no change in expression in either tissues between BMI groups.

The sigmoid colon showed no change in the mRNA expression of the genes assessed, between the two BMI groups (Figure 1F–J).

### 3.2. The Expression of PYY and Serotonin in Enterochromaffin and L-Cells Is Unchanged between Healthy BMI (<25) and Overweight/Obese BMI (≥25)

**Figure 2:**
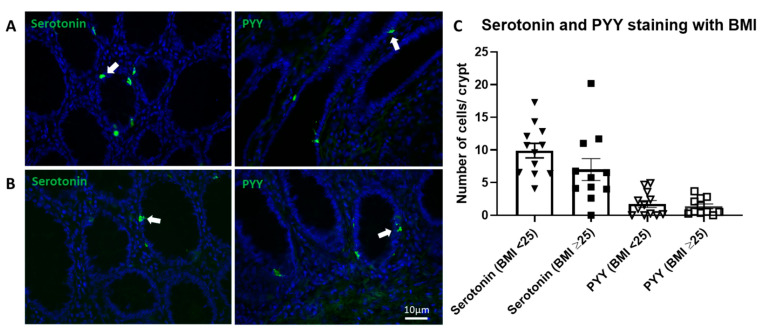
Serotonin and PYY immunohistochemistry expression in human colonic tissue of BMI < 25 and ≥25. (**A**,**B**): Representative images of serotonin and PYY expression in patients of BMI < 25 and BMI ≥ 25, respectively. Arrows denote serotonin and PYY positive cells. (**C**): Counts of cells per crypt stained positively for serotonin and PYY, grouped as BMI < 25 (*n* = 12) and BMI ≥ 25 (*n* = 11). Scale bars represent 10 μm.

In colonic tissue samples (from the Royal London Hospital, London, UK) serotonin containing EC cells were present in colonic crypts in both the <25 and ≥25 BMI groups (Figure 2A,B, respectively). PYY positive L-cells were also present in colonic crypts in both BMI groups (Figure 2A,B). The quantification of EC and PYY cells showed no significant difference between BMI groups (Figure 2C).

### 3.3. mRNA Expression of GPR40 Is Significantly Increased in Sigmoid Colon of ≥25 BMI Group

**Figure 3:**
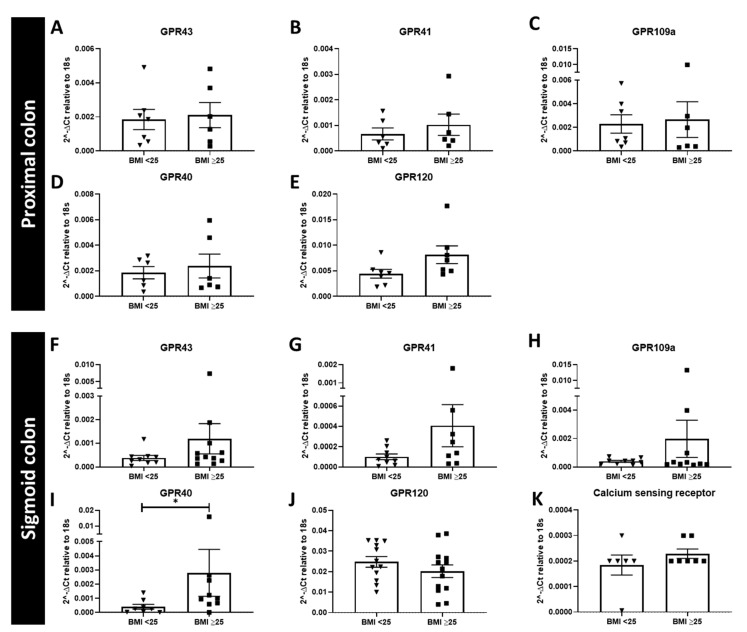
Relative mRNA expression of genes for nutrient receptors in the proximal and sigmoid colonic tissue from a Dutch cohort. Relative expression (against 18s) of nutrient receptors in the proximal (**A**–**E**, *n* = 6–7) and sigmoid colon (**F**–**K**, *n* = 6–13) with patients divided into those with a BMI < 25 and BMI ≥ 25, respectively, * significant increase.

In the proximal colon there was no significant difference in mRNA expression between BMI groups for the SCFA receptors GPR43, GPR41 and GPR109a (Figure 3A–C). There was no significant difference in mRNA expression of the LCFA receptor GPR120 (Figure 3E) and the M/LCFA receptor GPR40 (Figure 3D).

In the sigmoid colon, there was no significant difference in the mRNA expression of GPR43, GPR41, GPR109a, GPR120, or CaSR (Figure 3F–K). mRNA expression of GPR40 was significantly increased in the BMI ≥ 25 compared to BMI < 25 group (*p* = 0.0464) (Figure 3I).

**Figure 4:**
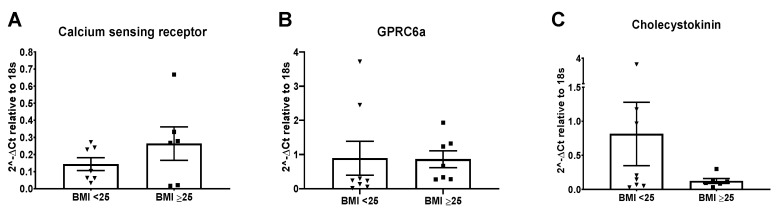
Relative mRNA expression of nutrient receptors in colonic tissue from a UK cohort. Relative expression (against 18s) of (**A**) CaSR (*n* = 7; *n* = 6), (**B**) GPRC6a (*n* = 8; *n* = 7) and (**C**) cholecystokinin (*n* = 8; *n* = 6) in a cohort of patients from the Royal London Hospital, divided into those with a BMI < 25 and BMI ≥ 25, respectively.

Individual qPCR expression experiments were conducted on proximal colonic tissue obtained from patients attending the Royal London Hospital to assess mRNA levels that were at a low detection level in samples from Maastricht. There was no significant difference in the expression of the amino acid sensing GPCRs, CaSR or GPRC6A in the BMI groups assessed (Figure 4A,B). Expression of the anorectic hormone CCK was also not significantly changed between the two BMI groups assessed (Figure 4C).

### 3.4. CaSR Is Highly Expressed on Serotonin Positive ECs Irrespective of BMI

**Figure 5:**
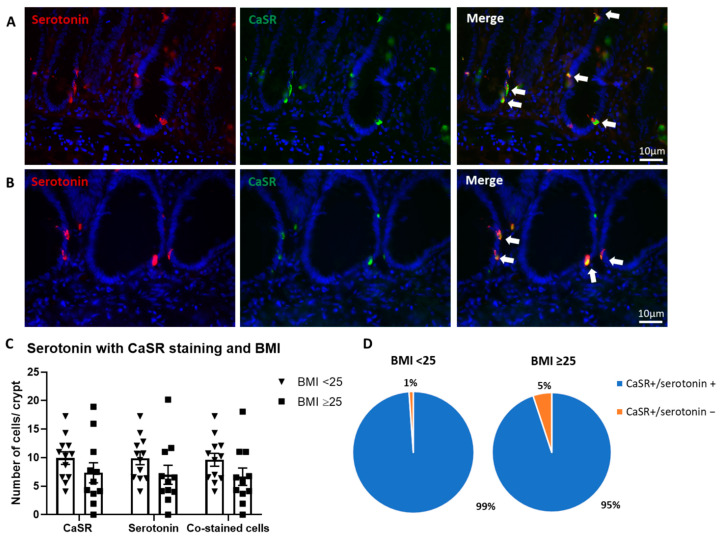
Serotonin and CaSR expression in human colonic samples according to BMI. (**A**): Representative image from BMI < 25 patient tissue staining for serotonin (red) and CaSR (green). (**B**): Representative image BMI ≥ 25 patient tissue staining for serotonin (red) and CaSR (green). Arrows denote serotonin and CaSR co-stained cells. (**C**): Quantification of positive cells per crypt for CaSR, serotonin and co-stained cells in BMI < 25 (*n* = 12) and BMI of ≥25 (*n* = 11). (**D**): Proportion of cells per crypt CaSR+/serotonin+ or CaSR+/serotonin- in patients with a BMI < 25 and BMI ≥ 25. Scale bars represent 10 μm.

Serotonin containing EC cells co-stained with CaSR in both BMI groups (Figure 5A,B). Cell counting demonstrated no significant differences in individual CaSR or serotonin positive cells, or cells that were co-stained in the two BMI groups (Figure 5A,B). CaSR was expressed on 99% and 95% of serotonin containing EC cell in the normal BMI and obese/overweight groups, respectively (Figure 5C).

### 3.5. Limited Expression of CaSR on PYY Expressing L-Cells

**Figure 6:**
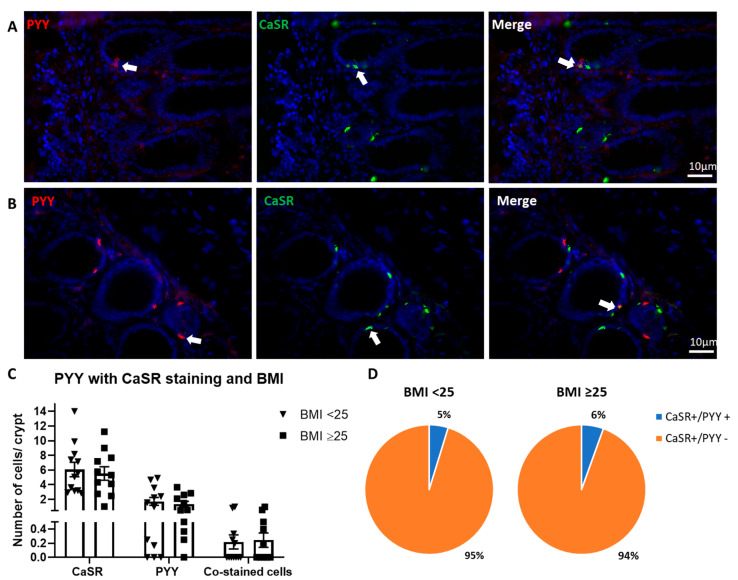
Serotonin and CaSR immunohistochemistry expression in human colon. (**A**): Representative image from BMI < 25 patient tissue staining for PYY (red) and CaSR (green). (**B**): Representative image BMI ≥ 25 patient tissue staining for serotonin (red) and CaSR (green). Arrows denote PYY and CaSR co-stained cells. (**C**): Number of cells per crypt for CaSR, PYY and cells co-stained with both PYY and CaSR, samples differentiated as BMI < 25 (*n* = 12) and BMI ≥ 25 (*n* = 11). (**D**): Proportion of cells per crypt that are CaSR+/serotonin+ or CaSR+/serotonin- in patients with a BMI < 25 and BMI ≥ 25. Scale bars represent 10 μm.

PYY and CaSR showed positive staining in our colonic tissue samples from both the healthy and overweight/obese groups (Figure 6A,B, respectively). Quantification of positively stained cells showed no significant changes in the expression of CaSR, PYY, or co-stained cells between healthy or overweight/obese BMI groups (Figure 6C). Co-staining of PYY with CaSR was infrequent—5% and 6% of cells in the healthy BMI and obese/overweight BMI groups, respectively (Figure 6D).

## 4. Discussion

We show that overall, overweight/obesity does not alter the gene expression of hormones/peptides, nutrient-sensing GPCRs for SCFAs, the LCFA GPR120, or amino acids. Similarly, at the protein level, the expression profile of EC and L cells is unchanged between healthy and overweight/obese BMI groups. However, we do show a modest increase in expression of the medium and long chain fatty acid receptor GPR40 in the sigmoid colon of patients in the BMI ≥ 25 group.

We report that both PYY mRNA expression and the number of PYY containing L cells are unchanged with increasing BMI. Our data suggests that the decreased circulating levels of PYY described previously [24,25] are not caused by reduced PYY mRNA expression or L-cell numbers, indicating that PYY release may be impaired. In addition, the mRNA expression of glucagon (a marker of GLP-1 production) is not significantly different between the two BMI groups, in either the sigmoid or proximal colon. GLP-1 and PYY have been shown to be co-expressed and co-released from secretory vesicles in L cells [23] therefore, it is expected that their release profile will be similar. Indeed, it has been demonstrated that obese individuals have decreased PYY and GLP-1 plasma levels that persisted for at least 12 months, despite weight loss [53]. Taken together with our data, this suggests that while the cells and receptors regulating cell function are unchanged in obesity, the cellular mechanisms leading to hormone release may be altered.

EC cells-mediated release of serotonin has been shown to be increased in obese vs. non-obese patients but the colonic mRNA expression of TPH1, an enzyme critical for serotonin production, is unchanged [56]. We similarly report no change in the expression of TPH1 and show that EC numbers in human colon do not change in response to BMI. These findings concur with a mouse study where EC numbers were unchanged in the jejunum between high and low fat diet animals [9]. However, our group has previously shown that expression of TPH-1 and the numbers of ECs were increased in the colons of an obese mouse group compared to a wild-type group (though this increase did not reach statistical significance) [49]. Collectively, these studies demonstrate that expression of TPH1 mRNA and serotonin containing EC cells are stable in humans despite weight changes. Furthermore, there may be changes at the genetic and protein levels in the murine colon, compared to the human colon.

Other hormones, such as the anorexigenic hormones leptin and CCK, were unchanged between the low and high BMI groups, as was the mRNA expression of somatostatin. Leptin, produced by adipose tissue, is increased in obesity (in serum and plasma) [57]. Our data shows that the colonic tissue has no impact on leptin changes. Unchanged levels of CCK may reflect the small population in the colon, as I cells expressing CCK are found primarily in the small intestine [31]. Somatostatin containing D cells are commonly found in the duodenum and stomach, while colonic populations constitute 3–5% of cells [34]; therefore, changes to somatostatin expression may occur primarily outside of the colon.

mRNA expression of nutrient-sensing GPCRs which activate and stimulate release of the cells described were similarly unchanged overall. However, there was a modest increase in GPR40 mRNA expression in the sigmoid colon of the overweight/obese BMI group. No change in GPR40 expression was observed in the proximal colon despite the previously reported equal distribution between the proximal and distal colon [45]. GPR40 binds to medium/long chain fatty acids and is expressed on human L cells, with agonist action increasing the release of GLP-1 [58]. The difference between the proximal and sigmoid colon expression of GPR40 may be attributed to its expression on L-cells, as density of these cells increases along the colon, with highest levels found in the rectum [18]. Therefore, it may be that greater numbers of cells expressing GPR40 are found in the sigmoid region, accounting for the small difference in expression observed. GPR120, another important MCFA sensing GPCR, was also unchanged in our study. This was a surprising result as absence of the gene encoding GPR120 induces an obese phenotype in humans [59] and mRNA expression is reportedly increased in the duodenum of obese and overweight humans [60]. However, in diet-induced obese mice, our group has previously shown a significant increase in mRNA expression of GPR120 and GPR40 between in obese mice [49]. Our data suggests that the colonic expression of GPR120 is stable and less likely to be influenced by increased caloric intake as most nutrients are absorbed in the small intestine.

Although reports suggest that the microbiome is altered in obesity [61] and that the luminal concentration of by-products of bacterial fermentation, i.e., SCFAs, may be altered [62], we report no change in the mRNA expression of the SCFA receptors GPR43, GPR41 or GPR109a. GRP43 and GPR41 knock-out mice have impaired L cell activity [63,64] suggesting that these receptors are involved in peripheral mechanisms of satiety and we have previously shown significant increase in expression of GPR41 or GPR43 in diet-induced obesity [49]. However, data from this study in human colonic tissue suggests that while the luminal environment may be altered in terms of bacterial populations and subsequent fermentation products, the expression of SCFA GPCRs remains stable.

To understand whether changes to GPCR and EEC expression patterns were affected by increasing BMI, we assessed co-expression via immunofluorescence. CaSR and EC cells were highly co-localised while a small population of PYY containing L-cells also expressed CaSR in human colonic tissue, regardless of BMI. We have previously reported a similar expression pattern of CaSR with PYY and serotonin in human colonic tissue [45]. Our data shows that obesity (BMI > 25) does not alter the expression of CaSR on EEC types. Activation of CaSR expressed on EECs has been shown to release gastrin, CCK, and GLP-1 [47,65] while L-cells from CaSR deficient mice show impaired release of CCK and Ca^2+^ in response to L-phenylalanine [47], suggesting that CaSR is crucial for their release. Our data suggests that agonists of CaSR may be an important target in obesity, as there is conservation of the cellular pathways which may be targeted for L-cell activation.

An important limitation of the current study is that the release of hormones from EECs cells or the ability of cells to become activated by nutrients binding to their corresponding nutrient receptors was not assessed. Clinical data has shown that circulating levels of these hormones is altered with obesity, therefore it is likely that hormone release is abrogated from these cells [24,25]. Importantly, maintenance of long-term satiety is critically dependent on PYY and GLP-1 [22,66]. Therefore, further studies are required to understand the capacity of EC and L cells from overweight/obese individuals to release their hormone content in response to nutrient stimulation. The focus of this study was the peripheral mechanisms regulating appetite in order to understand changes to molecular machinery in response to obesity. Lack of changes to expression of nutrient-sensing receptors and EECs does not exclude possible changes to downstream pathways in the periphery, including changes to hormone release profiles and afferent nerve activity, and warrants further investigation.

Overall, this study demonstrates that in human colonic tissue, the expression of nutrient sensing GPCRs, satiety hormones and EEC does not significantly change in the overweight/obese state. As previously discussed, obesity is characterised by an increased caloric load, changes to the microbial and luminal environment in obesity. However, our findings indicate that the expression pattern of the receptors and cells that sense luminal content is stable in the human colon.

## Figures and Tables

**Table 1:** Inclusion and exclusion criteria for counting of stained cells.

Inclusion Criteria	Exclusion Criteria
Whole cells	Parts of cells visible within fields of view (FOV)
Cells clearly distinguishable with nucleus (shape, size)	Ambiguity in defining cell shape
Cells located within crypts	Cells located outside of crypts
Levels of background low	High background/noise across the FOV

## Data Availability

All data relevant to this study is presented here or in the supplementary data associated with this publication.

## Supplementary Materials

The following are available online at https://www.mdpi.com/article/10.3390/nu13041271/s1, Graphs S1: Distribution of BMI for patient samples from the Royal London Hospital, UK. BMI data from individuals (*n* = 32) from whom colonic tissue was collected is plotted alongside their assigned patient number., Graphs S2: Distribution of BMI for patient samples from the University of Maastricht. BMI data from individuals (*n* = 67) from whom colonic tissue was collected., Table S1: Patient demographics from the Royal London Hospital, U.K., Table S2: Details of patient samples recruited at the University of Maastricht, Netherlands., Table S3: Primary antibodies used for immunohistochemistry, Table S4: Secondary antibodies used for immunohistochemistry, Table S5: Primers for gene expression studies, Table S6: Distribution of patient samples by gene examined by Taqman qPCR.
