# Supplementary material for: Effect of Obesity on the Expression of Nutrient Receptors and Satiety Hormones in the Human Colon
Source: Nutrients. 2021 Apr 13;13(4):1271. doi: 10.3390/nu13041271 (PMC8070384; doi:10.3390/nu13041271)
Supplement: Supplementary file 1 [file nutrients-13-01271-s001.pdf]

Supplementary Data

Supplementary Table 1: Patient demographics from the Royal London Hospital, U.K.

	Female		Male	
**N: (%)**	10	(33%)	20	(67%)
**Age: mean, +/- SD (years)**	62.4	±19.2	61.5	±18.1
**BMI: mean,+/- SD**	26.2	±4.1	25.7	±5.6

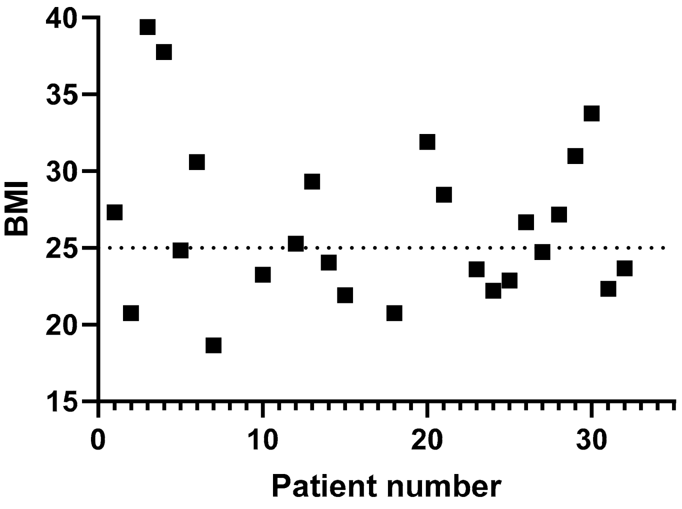

Supplementary Graph 1: Distribution of BMI for patient samples from the Royal London Hospital, UK. BMI data from individuals (*N*=32) from whom colonic tissue was collected is plotted alongside their assigned patient number.

Supplementary Table 2: Details of patient samples recruited at the University of Maastricht, Netherlands.

	Female		Male	
***N*: (%)**	47	69.1%	21	30.9%
**Age: mean, +/- SD (years)**	44.8	±16.2	34.7	±8.9
**BMI: mean,+/- SD**	25.8	±5.2	24.3	±5.2
**IBS subtype: *N*, (%)**				
**Constipation**	18	38.3%	0	0%
**Diarrhoea**	11	23.4%	2	9.5%
**Mixed**	15	31.9%	9	42.9%
**None (healthy control)**	3	6.4%	10	47.6%

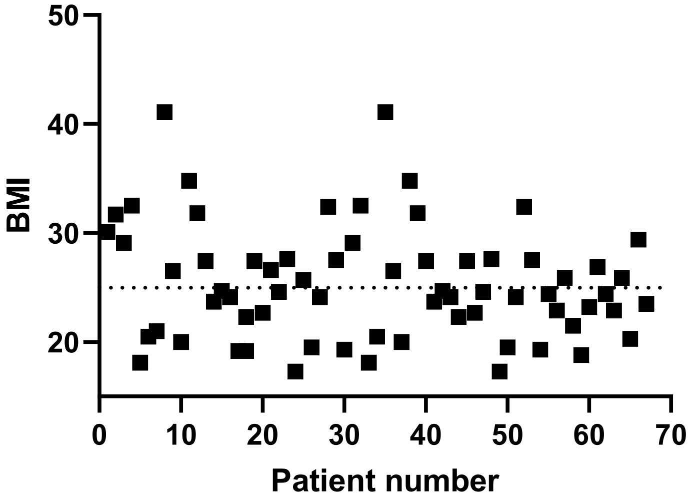

Supplementary Graph 2: Distribution of BMI for patient samples from the University of Maastricht. BMI data from individuals (*N*=67) from whom colonic tissue was collected.

Supplementary Table 3: Primary antibodies used for immunohistochemistry

Protein	Species	clonal	Company	Product ID	Dilution
CaSR	Mouse	mono	Abcam	AB-19347	1:200
PYY	Rabbit	poly	US Biological	P3285-10	1:200
5-HT	Goat	poly	Immunostar	20079	1:200

Supplementary Table 4: Secondary antibodies used for immunohistochemistry

Species	Species reactivity	Wavelength	Company	Product ID	Dilution
Chicken	Mouse	488	Invitrogen	A21200	1:400
Donkey	Rabbit	568	Invitrogen	A10042	1:400
Donkey	Rabbit	488	Invitrogen	A21206	1:400
Donkey	Goat	568	Invitrogen	A11057	1:400
Donkey	Goat	488	Invitrogen	A11055	1:400

Supplementary Table 5: Primers for gene expression studies

Gene/ target	Manufacturer	Product code
Cholecystokinin	Qiagen	QT00073871
Cholecystokinin vb (CCK B)	Qiagen	QT02405228
Cholecystokinin va (CCK A)	Qiagen	QT02405221
Calcium-sensing receptor	Qiagen	QT00055944
GPRC6a	Qiagen	QT01033102
IFN-y	Qiagen	QT00000525
18s ribosomal RNA	Qiagen	QT00199367

Supplementary Table 6. Distribution of patient samples by gene examined by Taqman qPCR.

**Proximal**		**Sigmoid**
A (n = 6, n = 6)	GPR43	F (n = 9, n = 11)
B (n = 6, n = 6)	GPR41	G (n–9, n = 8)
C (n = 7, n = 6)	GPR109a	H (n–9, n = 10)
D (n = 6, n = 6)	GPR40	I (n–9, n = 8)
E (n = 7, n = 7)	GPR120	J (n = 12, n = 13)
	Calcium sensing receptor	K (n = 6, n = 7)
